# Observational evidence confirms modelling of the long-term integrity of CO_2_-reservoir caprocks

**DOI:** 10.1038/ncomms12268

**Published:** 2016-07-28

**Authors:** N. Kampman, A. Busch, P. Bertier, J. Snippe, S. Hangx, V. Pipich, Z. Di, G. Rother, J. F. Harrington, J. P. Evans, A. Maskell, H. J. Chapman, M. J. Bickle

**Affiliations:** 1 Shell Global Solutions International, Kessler Park 1, 2288 GS Rijswijk, The Netherlands; 2 Department of Earth Sciences, University of Cambridge, Downing Street, Cambridge CB2 3EQ, UK; 3 Clay and Interface Mineralogy, RWTH Aachen University, Bunsenstrasse 8, 52072 Aachen, Germany; 4 High Pressure and Temperature Laboratory, Utrecht University, PO Box 80 021, 3508 TA Utrecht, The Netherlands; 5 Jülich Centre for Neutron Science (JCNS), Forschungszentrum Jülich GmbH, Outstation at Heinz Maier-Leibnitz Zentrum (MLZ),1 Lichtenbergstrasse, 85747 Garching, Germany; 6 Chemical Sciences Division, Oak Ridge National Laboratory, Oak Ridge, Tennessee 37831, USA; 7 British Geological Survey, Environmental Science Centre, Keyworth, Nottingham NG12 5GG, UK; 8 Department of Geology, Utah State University, 4505 Old Main Hill, Logan, Utah 84322-4505, USA

## Abstract

Storage of anthropogenic CO_2_ in geological formations relies on a caprock as the primary seal preventing buoyant super-critical CO_2_ escaping. Although natural CO_2_ reservoirs demonstrate that CO_2_ may be stored safely for millions of years, uncertainty remains in predicting how caprocks will react with CO_2_-bearing brines. This uncertainty poses a significant challenge to the risk assessment of geological carbon storage. Here we describe mineral reaction fronts in a CO_2_ reservoir-caprock system exposed to CO_2_ over a timescale comparable with that needed for geological carbon storage. The propagation of the reaction front is retarded by redox-sensitive mineral dissolution reactions and carbonate precipitation, which reduces its penetration into the caprock to ∼7 cm in ∼10^5^ years. This distance is an order-of-magnitude smaller than previous predictions. The results attest to the significance of transport-limited reactions to the long-term integrity of sealing behaviour in caprocks exposed to CO_2_.

## Abstract (web summary)

Anthropogenic CO_2_ storage, where CO_2_ is injected into saline geological resevoirs, relies on an impermeable caprock to seal in the CO_2_, but caprock reaction rates to CO_2_ acid brines are unclear. Here, Kampman *et al*. show that mineral reaction front alteration in caprocks takes place over 100,000 years.

## Graphical abstract

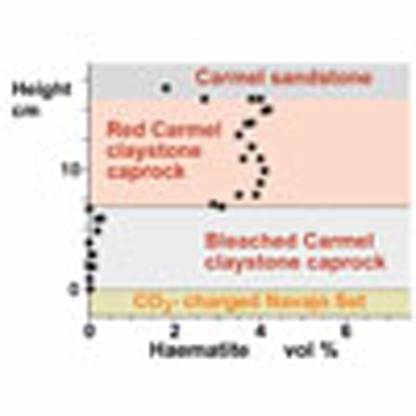

Carbon capture and storage will form an essential part of the technologies needed to reduce anthropogenic CO_2_ emissions if costly climate change is to be avoided^1^. CO_2_, separated at power stations and industrial plants, is compressed and injected into saline geological reservoirs as a supercritical fluid^2^. The CO_2_ is less dense than the saline brines at typical formation temperatures, driving buoyant migration, and will need to be retained by impermeable caprocks as the primary mechanism for ensuring effective storage. Subsidiary processes including dissolution of CO_2_ in formation brines, trapping of CO_2_ by capillary forces and precipitation of carbonate minerals will aid storage security over the 10^4^ year timescales needed to avoid climate impacts^3^, but operation of these processes is dependent on the initial retention under the caprock. The caprocks in sedimentary successions suitable for CO_2_ storage will most commonly comprise clay-rich mudstones or shales that act as barriers to CO_2_ migration by two mechanisms^4^. Their low permeabilities (<10^−19^ m^2^) restrict fluid fluxes to very low rates. Further, capillary entry pressures for CO_2_ of 0.5–5 MPa^4^ will restrict penetration of CO_2_, allowing transport of CO_2_ only by sluggish diffusion in the brine phase, which will be further retarded by the complex geometry of the pore networks. The addition of CO_2_ forms acid brines that react with clay-rich caprocks to drive dissolution and precipitation of minerals^5^,^6^,^7^, with consequences for the evolution of porosity and the geomechanical integrity of the seals^8^, within regions penetrated by the diffusing CO_2_. The initial dissolution of rapidly reacting carbonate and oxy-hydroxide phases will buffer the pH of CO_2_-saturated brines to ∼5 followed by the more sluggish dissolution of silicate and phyllosilicate minerals, which further increase pH and cause re-precipitation of carbonate phases^5^,^9^,^10^. However, it is uncertain as to whether these fluid–rock reactions may lead to mineral precipitation-induced self-sealing phenomena that limit the pervasion of the CO_2_, as predicted by some numerical simulations^5^,^9^,^10^,^11^,^12^,^13^, or self-enhancing mineral dissolution and porosity generation, which generate a continuous increase in caprock transport properties, as observed in some laboratory experiments^14^,^15^,^16^,^17^. Direct observations of caprock materials exposed to CO_2_ over the timescales requisite for storage are critical to address this uncertainty, where the coupled reactive-transport phenomena are integrated over the appropriate temporal and spatial scales.

Fundamental uncertainties in the prediction of coupled reactive transport in low permeability clay-rich caprocks include a poor understanding of the relationship between reaction-induced changes in porosity and the pore-network structure, and the consequences for the rates of solute transport^18^, the uncertain mineral surface areas and kinetics of the mineral reactions in natural settings^19^, the rates of which may be limited by the intrinsic surface reaction rates of the minerals or by rates of solute transport^20^, and the reaction pathways in natural settings, including the role of acid redox reactions as a CO_2_ sink^21^, which will retard the rates of diffusive CO_2_ transport.

We address this fundamental gap in our knowledge of the long-term impacts of CO_2_-charged fluids on caprock integrity by examining caprock recovered from a natural CO_2_ reservoir. Although this reservoir is at much shallower depths and lower pressures than needed for geological carbon storage, the reactions between minerals and CO_2_-saturated brines are little affected by pressure. Mineralogical and petrophysical measurements are used to assess the impacts of CO_2_-promoted reactions on the caprock pore-network transport properties. The measurements reveal that the CO_2_-charged mildly reducing brines only removed haematite from the basal 7 cm of the haematite-bearing claystone cap rock. This data is used to constrain analytical and numerical reaction–diffusion models, which confirm that the fluid–rock reactions retard CO_2_ transport by an order of magnitude, and that the timescale of alteration is ∼10^5^ years, comparable with geochronological constraints on the duration of CO_2_ storage in the reservoir. The small but transient increases in porosity generated by the mineral reactions do not significantly enhance reaction front velocities. The results attest to the significance of transport-limited reactions to the long-term integrity of sealing behaviour in caprocks exposed to CO_2_.

## Results

### Mineral reactions and petrophysical changes in the caprock

**Figure 1:**
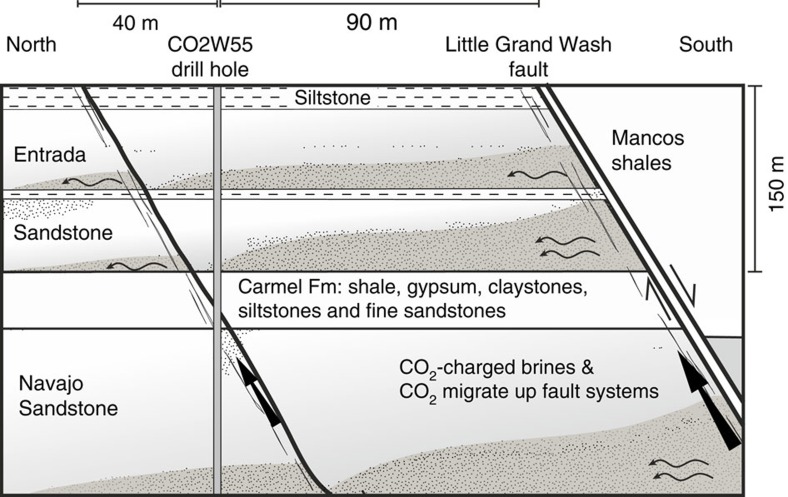
Geological setting of the CO2W55 drill hole. The hole was drilled 90 m north of the Little Grand Wash Fault south of Green River, Utah^22^,^23^. It transected the Entrada Formation, the Carmel Formation, a small splay fault related to the Little Grand Wash fault within the Carmel Formation, and entered the Navajo Formation at a depth from surface of 200.11 m. Dissolved CO_2_ and salinity gradients within fluids sampled from the Navajo Formation indicate that CO_2_-saturated brines (shaded) migrate (wavy lines) from the fault zones along the base of the aquifers and mix with meteoric groundwaters^23^. Horizons with similar CO_2_-charged fluids were encountered in the Entrada and Carmel Formations.

Core and fluid samples were collected from a sequence of CO_2_-charged reservoirs and intervening caprocks during scientific drilling within the footwall of the Little Grand Wash Fault, Green River, Utah^22^ (Fig. 1). The 325-m deep drillhole transected reservoirs of CO_2_-charged brine in the Middle Jurassic Entrada and Lower Jurassic Navajo Sandstone, and the intervening Middle Jurassic Carmel Formation caprock. The geochemistry of fluid samples, collected at formation pressures through the Navajo Sandstone reservoir, tracks contemporary filling via artesian flow of CO_2_ and CO_2_-saturated brines through the faults^23^. The reservoir fluids are mixtures of Na-Cl-SO_4_ brine and meteoric groundwater with low pH (5.1–5.3), reducing (0 to −50 mV), SO_4_-rich and contain trace quantities of H_2_S and CH_4_. The migrating CO_2_ and CO_2_-saturated brine are thought to originate from an accumulation of supercritical CO_2_ within Carboniferous strata at depth^24^. U-Th geochronology of carbonate veining in associated faults attests to CO_2_ out-gassing regionally for *ca*. 400 ka and locally for at least 114 ka, providing an independent constraint of the duration over which the Carmel Formation caprock has been exposed to CO_2_-charged brines^25^. Cycles in the chemistry of the fault-hosted carbonate veins have been attributed to cyclic charging of the shallow reservoirs with multiple pulses of CO_2_-rich fluids, which coincide with periods of crustal unloading during regional deglaciations^26^.

The Carmel Formation constitutes a regional seal between the Navajo and Entrada aquifers. It comprises a 50-m-thick complex package consisting of three major lithofacies; interbedded, unfossiliferous red and grey shale and bedded gypsum, red and grey claystone/siltstone and fine-grained sandstone^23^. These are interpreted as marine sediments deposited in quiet, subtidal conditions under the influence of periodic hypersaline water^27^.

**Figure 2:**
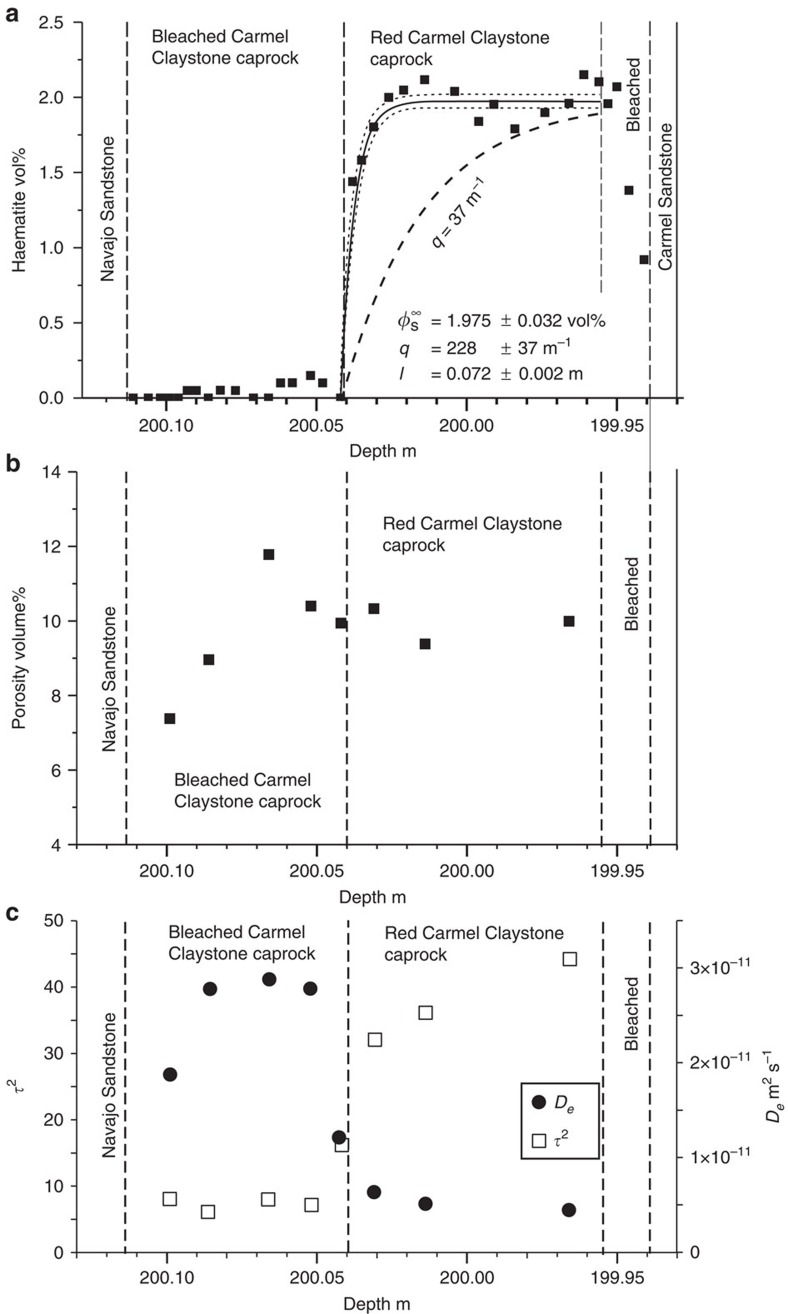
Mineral profiles across Caprock. (**a**) Haematite (vol-%) profiles across the basal Carmel claystone. Solid curve is least-squares best fit to haematite vol-% profile to equation (6) adjusting *ϕ*_s_, *q* and *l* (best fit values and 1*σ* uncertainties given on figure) with 1*σ* uncertainty on haematite of 7% relative, but a minimum uncertainty of 0.1 volume %. Dashed line shows profile for *q*=37 m^−1^ taken as lower limit as discussed in text. It is noteworthy that the upper 1 cm of the claystone is bleached adjacent to a CO_2_-bearing sandstone within the Carmel Formation. (**b**) Porosity calculated from SANS/VSANS measurements across caprock. (**c**) Tortuosity, *τ*^2^, (left-hand axis) and diffusivity *D*_e_, (right-hand axis) calculated from SANS/VSANS measurements across caprock (see Methods).

In the Green River drill hole, a basal 16-cm-thick claystone of the Carmel Formation acts as a caprock to the CO_2_-rich brines in the underlying Navajo Sandstone. This claystone was subsampled at ∼4 mm vertical resolution and analysed for quantitative mineralogy, carbonate *δ*^18^O and *δ*^13^C isotopic compositions, ^87^Sr/^86^Sr ratios, bulk mineral surface area and porosity (Fig. 2a, Methods, Supplementary Fig. 1 and Supplementary Tables 1–3).

The primary mineralogy of the unaltered portion of the red claystone comprises illite, quartz, dolomite, K-feldspar and haematite (Supplementary Fig. 2), with >50% clay-sized particles. This basal claystone is typical of oxidized redbed shallow marine and continental mudstones, forming a representative analogue to other important reservoir seals such as the North Sea Mercia Mudstone^28^ and the Formations in the Triassic Keuper^29^.

The basal 7 cm of the originally red claystone, at the caprock-reservoir interface, is distinctively bleached white–yellow, reflecting quantitative removal of haematite (Fig. 2a), with loss of the primary dolomite and precipitation of ankerite–dolomite, sulphide minerals and gypsum (Fig. 2a and Supplementary Fig. 1A). Carbonate, sulphate, sulphide and clay mineral compositions have been analysed using Electron microprobe analyses (EMPA; Methods and Supplementary Table 4). Large-scale bleaching of exhumed redbed sandstones and siltstones in the Green River location has previously been related to flow of reducing CO_2_-bearing fluids^30^,^31^,^32^. A comparable basal unit from the Carmel Formation from a drill hole free from CO_2_ 33 km north west (NW) of Green River shows no discernible change in mineralogy or O- and C-isotope ratios as a result of reactions at the base of the caprock (Supplementary Fig. 3).

The observed petrological changes are consistent with a series of interdependent reactions involving CO_2_- and H_2_S-promoting dissolution of haematite following the stoichiometry


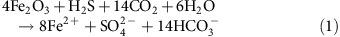


Dolomite dissolution was coupled with the growth of ankerite–dolomite, with Fe^2+^ derived from the acid-reductive dissolution of haematite (Fig. 2a and Supplementary Fig. 2A,B), accompanied by shifts in the O-, C- and Sr-isotopic composition of the newly precipitated ankerite–dolomite cements (Supplementary Fig. 1B–D). Pyrite precipitated upstream and euhedral haematite, chalcopyrite and covellite precipitated downstream of the haematite dissolution front, with Fe and Cu supplied from primary haematite dissolution and sulphide supplied from the invading fluid (Supplementary Fig. 2B–D). The K-feldspar dissolution close to the reservoir caprock interface led to the precipitation of pore-filling illite (Supplementary Fig. 2E,F).

Pore network structures at 4 mm intervals along the reaction profile were characterized using N_2_-BET and small and very small angle neutron scattering techniques ((V)SANS; see Methods). The porosity profile shows a remarkable increase in porosity directly upstream of the haematite dissolution front, but further upstream precipitation of ankerite–dolomite, pyrite, gypsum and illite cause a progressive decrease in porosity to values below that of unreacted caprock (Fig. 2b and Supplementary Table 3).

The tortuosity (*τ*^2^) of the pore network at each sample point in the profile (Fig. 2c) was calculated from the surface and volume fractal dimensions of the pore structure, measured by the (V)SANS data (Supplementary Fig. 5 and Methods). This allows the calculation of the effective diffusivity of a species, *D*_e_ (m^2^ s^−1^) in the tortuous pore network, which is related to the diffusion coefficient in water, *D*_w_, and the diffusion accessible porosity, *ϕ*, by


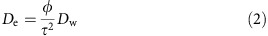


The molecular diffusivity of CO_2_ and H_2_S in water at 20 °C is ∼2 × 10^−9^ m^2^ s^−1^ (ref. 33). The calculated porosities, surface fractal dimensions, *D*_s_, and pore volume fractal values, *D*_v_, (Supplementary Table 3) imply a *τ*^2^ of ∼37 and effective diffusivities for CO_2_ and H_2_S of 5 × 10^−12^ m^2^ s^−1^ in the unaltered portions of the caprock (Fig. 2c). The calculated porosities agree well with the porosity distribution measured by N_2_-BET over the same size range for sample NPS-069 (Supplementary Fig. 6). Clarkson *et al*.^34^ and Bertier *et al*.^35^ discuss comparison of porosities calculated from (V)SANS data with N_2_ physiosorption data for comparable shales and emphasize the limitations of the physiosorption data. The calculated diffusivities are at the lower limits of experimentally determined measurements for tortuosity and CO_2_ effective diffusivities in shales^18^,^36^,^37^,^38^, and are consistent with the relative high clay content of these samples. It should be noted that our calculations assume that the porosity is interconnected for pores over the length scales sampled (1.2–1,500 nm).

In the altered portion of the caprock the estimated tortuosity decreases to 7 and effective diffusivities increase to 3 × 10^−11^ m^2^ s^−1^, because pore connectivity increases and the roughness of the pore–solid interface decreases due to mineral dissolution (Fig. 2c). The diffusivities decrease to 1.9 × 10^−11^ m^2^ s^−1^ further upstream following mineral reprecipitation, but the tortuosity of the pore network does not significantly increase, suggesting that there is a non-recoverable impact of mineral dissolution on rock transport properties.

### Modelling reactive transport in the caprock

The mineralogical profiles record the propagation of mineral–fluid reaction fronts within the caprock lithology, driven by the upward diffusion of CO_2_ and H_2_S. Modelling of the reactive transport enables examination of the key processes governing the reactive diffusion of CO_2_ including the timescale and the ratio of transport rates to mineral reaction rates. Upward advective transport by fluids must be negligible given the low permeability of the unreacted caprock of ∼10^−22^ m^2^ (Methods), which equates to a hydraulic conductivity of ∼10^−15^ m s^−1^, and thus a Péclet number of ∼10^−3^ m, over the length scale of the alteration. An analytical solution to one-component diffusive transport with mineral dissolution rate described by linear kinetics^39^ captures the important physics and informs the essential approximations necessary for reactive transport modelling with multiple components. On initiation of diffusion, the reactant mineral volume fraction decreases until the phase is exhausted at the base of the caprock. From this time, *τ*_0_, a reaction front migrates downstream away from the base, where *τ*_0_ is given by,


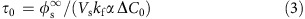


where *k*_f_ (m s^−1^) is mineral reaction rate, *α* (m^2^ m^−3^) is the mineral surface area, *V*_s_ is the mineral molar volume (m^3^ mol^−1^) and 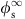 is the volume fraction of the reactant mineral phase initially present. *ΔC*_*0*_=*C*_eq_−*C*_0_ where *C*_eq_ is the solute concentration (mol  m^−3^) in equilibrium with the reactant mineral phase and *C*_0_ is the solute concentration in the infiltrating fluid (for full derivation of equations after ref. 39, see Methods), which can be modelled in terms of consumption of CO_2_, H_2_S or Fe_2_O_3_, for which the solutions are identical and fixed by the stoichiometry of reaction (1). The position of the reaction front, *l* (m) at time *t* (s) is given by solution of


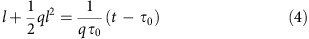


where *q* (m^−1^) is given by


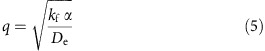


where *D*_e_ is the effective diffusion coefficient defined in equation (2). At *t*>*τ*_0_, the variation of reactant mineral volume with time and distance (*x*, *m*) downstream of the reaction front is given by


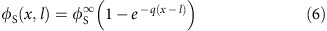


**Figure 3:**
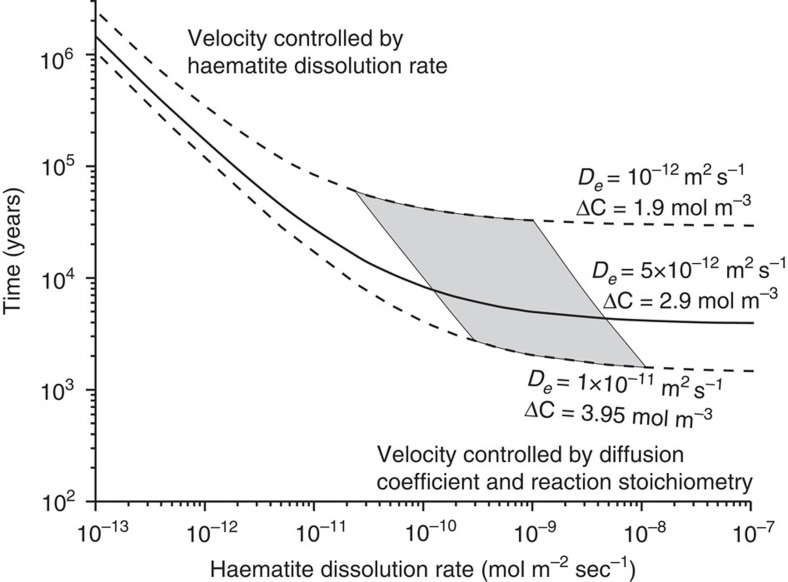
Solutions to the one-component transport equation. The duration of caprock alteration calculated from equation (4) given the time for the reaction front to develop (*τ*_0_; equation (3)) and the displacement distance of the front, *l*. Calculations are performed on the basis of Fe in equation (1). Shaded area denotes solutions for 37<*q*<228 m^−1^, which gives a haematite dissolution rate, *k*_R_, between 1 × 10^−10^ and 5 × 10^−9^ mol m^−2^ s^−1^ from equation (5) for a diffusion coefficient of 5 × 10^−12^ m^2^ s^−1^. Solid line shows median solution with *D*_e_=5 × 10^−12^ m^2^ s^−1^ and Δ*C*_0_=2.9 mol m^−3^. Dashed lines show extreme solutions with the minimum estimate of diffusivity of 10^−12^ m^2^ s^−1^ and minimum Δ*C*_0_ of 1.9 mol m^−3^ giving maximum reaction times and a high estimate of diffusivity of 1 × 10^−11^ m^2^ s^−1^, combined with a maximum estimate of Δ*C*_0_ of 3.95 mol m^−3^ giving minimum reaction times. Uncertainties in the duration of caprock alteration propagated from the other variables are small compared with those from the potential range of caprock diffusivities and reaction stoichiometry.

In the absence of advection, solutions for the position and velocity of the reaction front (equation 4) are divided into two regimes and the broadening of the front depends dominantly on the kinetics of the reaction^40^. If *ql*>1 (that is, the Damköhler number (*ql*)^2^ is large), reaction rates are fast compared with diffusion rates and the propagation rate depends on the diffusivity of the species driving the reaction and the reaction stoichiometry. For *ql*<1, the position of the reaction front is a linear function of time and the velocity of the front depends on the reaction rate constant and mineral surface area. Figure 3 illustrates such solutions, calculated for transport of Fe in the fluid phase, for time, *t*, as a function of haematite dissolution rate, *k*_R_ (mol m^−2^ s^−1^) given the reaction stoichiometry of equation (1) and contoured for the effective diffusivity of the caprock, *D*_e_, estimated from pore volume and fractal pore network modelling of SANS data.

The haematite mode profile (Fig. 2a) primarily reflects dissolution described by equation (1), but potentially with additional precipitation of haematite downstream of the reaction front as a result of multicomponent transport (Fig. 2a and *c.f.* ref. 30). The geometry of the mineral dissolution front, characterized by the spatial variation in haematite modes, is described by equation (6). The curvature of this profile, for an assumed constant effective diffusivity at and upstream of the dissolution front, depends only on the kinetics of the reaction. A least-squares best fit gives a value for the exponential constant *q* in equation (4) of 228±31 m^−1^ (1*σ*). If the profile is augmented by haematite precipitation, *q* might be as small as ∼37 m^−1^. Given that *l*=0.07 m (Fig. 2a), *ql* is >2 and reaction rates are fast compared with diffusive transport. For *D*_e_=5 × 10^−12^ m^2^ s^−1^ (Fig. 2c), the reaction stoichiometry of equation (1), a haematite-specific surface area calculated from a volume-weighted total SANS surface area and inlet fluid compositions from downhole sampling^23^ (Methods), these values of *q* imply a timescale between 3,000 and 10,000 years, and haematite dissolution rates in the range 1 × 10^−10^ to 5 × 10^−9^ m^2^ s^−1^ (Fig. 3). The latter compare well with laboratory estimates for haematite dissolution rates under acidic conditions in the presence of low concentrations of dissolved H_2_S^41^ (Methods).

**Figure 4:**
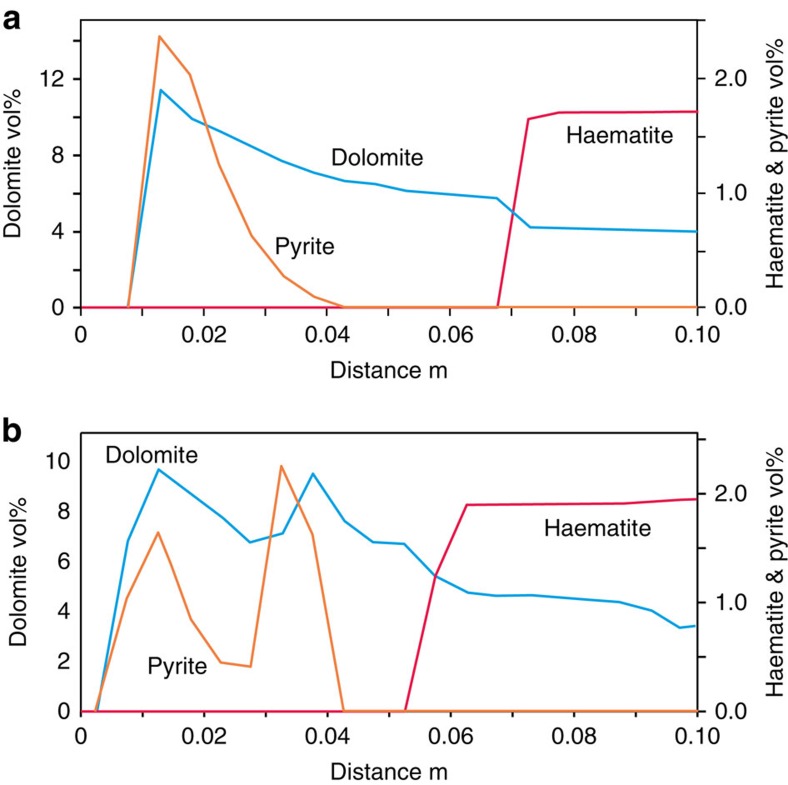
Diffusion-reaction modelling in PHREEQC. (**a**) PHREEQC^42^ model run for 125,000 years for constant CO_2_ at saturation on basal boundary. A 15 cm-long one-dimensional reactive–diffusive model comprising 30 cells of 5 mm length was used with initial mineralogy (mol l^−1^) calculated from XRD analyses, pore volumes from SANS of the unaltered portion of the caprock, the invading pore fluid chemistry based on the reservoir fluids (Supplementary Table 4) and the initial caprock pore fluid chemistry was taken to be a fluid in equilibrium with the caprock mineralogy, with pCO_2_, pO_2_ and salinity estimates for typical Jurassic marine shales. The initial redox state of the invading fluid was defined using the SO_4_^2−^/H_2_S redox couple. Models were run assuming local fluid–mineral equilibrium and a constant D_*e*_ value of 5 × 10^−12^ m^2^ s^−1^ was used for all aqueous species. The model timescale was 125,000 years with a time step of 7 days. (**b**) One-dimensional model with the same starting conditions as **a** but with two 25,000 year phases of CO_2_-saturated brine in the basal cell with an intervening 75,000 year period where the basal cell contained a CO_2_-poor brine. The results of the two episodes of CO_2_ saturation are seen as double peaks in pyrite and dolomite modes as the CO_2_ from each pulse diffuses into and reacts with the caprock.

The modelling above only considers transport of Fe_2_O_3_ and dissolution of haematite as the major component buffering fluid pH and oxidation state. The mineralogical profiles (Fig. 2a and Supplementary Fig. 1A) show that dissolution/precipitation of dolomite, ankerite–dolomite, sulphate, sulphide, K-feldspar and illite also occur. The reactive diffusion was modelled with multiple components and phases by numerical simulation using PHREEQC^42^ (Fig. 4, Methods). A 15-cm-long one-dimensional reactive–diffusive model comprising 30 cells of 5 mm length was constructed. Each cell initially contained the mineral volumes measured by X-ray diffraction (XRD) and porosity measured by (V)SANS in unreacted portions of the caprock. The fluid chemistry occupying the boundary reservoir cell was based on analyses of the modern reservoir fluids^43^ with a redox state defined using the SO_4_^2−^/H_2_S redox couple (sample DFS00413 with SO_4_ 20.7 mmol and H_2_S 0.5 mmol). The initial caprock pore fluid chemistry was calculated similar to that in equilibrium with the caprock mineralogy (reactive minerals haematite, Mg-dolomite, K-feldspar and illite), with *p*CO_2_, *p*O_2_ and salinity typical of Jurassic marine shales^43^ (Supplementary Table 4). Models were run assuming local fluid–mineral equilibrium and pyrite, an Fe-bearing dolomite (Mg_0.9_Fe_0.1_CaCO_3_), and illite were allowed to precipitate (Supplementary Table 2). A *D*_e_ value of 5 × 10^−12^ m^2^ s^−1^ was used for all aqueous species. The model timescale was 125,000 years, with a time step of 7 days required to reach convergence of the numerical solution.

The models reproduce the key observations (Fig. 4a). The haematite dissolution front migrates ∼7 cm in >10^4^ years. Dolomite dissolves downstream of the front and Fe-dolomite is precipitated upstream of the reaction front (Supplementary Fig. 2B). Pyrite is precipitated initially close to the caprock-reservoir contact and migrates very slowly. K-feldspar dissolves only at the inlet, driving local precipitation of illite. The additional complexities observed with multiple zones of pyrite precipitation probably result from the episodic release of CO_2_-charged brines by the fault system^26^. Models run with two episodes of CO_2_ injection reproduce a similar pattern in which the diffusion of mineral profiles into the caprock preserves a record of the evolution of reservoir fluid compositions (Fig. 4b) apparent as double peaks in pyrite and dolomite.

## Discussion

This study provides evidence for the slow penetration rate of CO_2_-promoted fluid–rock reactions into clay-rich caprocks and the development of a stable reaction front without a significant positive feedback loop between porosity enhancement and the velocity of the front. SANS methods are effective at resolving micrometre-scale changes in pore network characteristics and diffusive transport properties, with the magnitude of the change of the latter being approximately half an order of magnitude. This provides evidence to refute observations from laboratory experiments, which posit a significant increase in the transport properties of caprocks following exposure to CO_2_-charged brines^14^, and CO_2_-charged brines and H_2_S^17^. Multicomponent numerical modelling of the reactive–diffusive transport reproduced the major mineralogical changes accompanying diffusion of the CO_2_, although detailed variations reflect the complex heterogeneity of natural rocks, and the cyclic variation in CO_2_ charging of the underlying reservoir. The important conclusion from the analytical and numerical modelling is that the timescale for the development of the 7-cm reaction front is ∼10,000 to 100,000 years, consistent with the geological evidence for the duration of the CO_2_ supply to the Little Grand Fault system^25^. Previous numerical models of reactive–diffusive transport of CO_2_ in clay-rich caprocks, unconstrained by observations from natural systems, have predicted the development of mineral alteration profiles over length scales of metres on a 10,000 year timescale^5^,^9^,^10^,^12^. In addition, these modelling studies did not consider redox reactions, which as demonstrated here, are an important sink for CO_2_ invading the caprock and contribute significantly to retardation of the CO_2_-diffusive transport. The results demonstrate that numerical models of reactive transport in low-diffusivity media, where mineral reaction kinetics do not limit reaction rates, successfully predict reactions and reaction rates, providing the full chemical complexity is modelled. The results also show that the mineralogy of caprocks and chemistry of potential CO_2_-charged brines will need to be considered on a case-by-case basis.

Fluid–rock reaction has substantially retarded diffusive transport of the CO_2_, which would have penetrated ∼8 m in 100,000 years in the absence of reaction, based on a diffusion coefficient of 5 × 10^−12^ m^2^ s^−1^. Rock-buffering reactions are effective at establishing fluid–mineral equilibrium over centimetre length scales. In this caprock, CO_2_-charged fluid–mineral reactions act to enhance caprock integrity over a time period comparable to that needed for effective geological carbon storage.

## Methods

### Drill core processing

HQ core (63.5 mm diameter) samples of the caprock interval, recovered from well CO2W55 near the town of Green River, Utah, were slabbed and then sliced parallel to bedding at ∼3–5 mm resolution, using a steel blade rock saw. Approximately 2–5 g of air-dried sample was powdered to ≤100 μm size in a ball mill using an agate container and balls, and subsamples were taken for individual analyses. Samples for XRD measurements were crushed and milled separately. A comparative sample profile from the base of the Carmel Formation, in a region where CO_2_ is absent, was collected from sections of core BH2 recovered during drilling of the Bighole fault^44^, located ∼35 km north east (NE) of Green River. This comparison profile was prepared and analysed for quantitative mineralogy and carbonate O- and C-isotopes using the same methods as for the CO2W55 core interval (Supplementary Fig. 3).

### XRD measurements

XRD measurements to determine mineralogy (Supplementary Table 1) have been performed at RWTH Aachen University as stated in ref. 45, except the counting time is 20 s for each step of 0.02° 2θ recorded from 2° to 92° 2θ. Rock samples are crushed manually in a mortar with care taken to avoid strain damage and crushed material together with an internal standard (Corundum, 20 wt%) is milled in ethanol with a McCrone Micronising mill (15 min). Quantitative phase analysis is performed by Rietveld refinement using BGMN software, with customized clay mineral structure models^46^. The precision of these measurements, from repetitions, is better than 0.1 wt% for phases of which the content is above 2%. The accuracy cannot be determined because of the lack of pure clay mineral standards, but is estimated to be better than 10% (relative). Mineral compositions relate to the crystalline content of the analysed samples.

### Stable isotopes

Carbonate mineral *δ*^13^C and *δ*^18^O were determined at the University of Cambridge on duplicate subsamples of 300–500 μg using a Thermo Gas Bench attached to a Thermo MAT 253 mass spectrometer in continuous flow mode, with an analytical precision of ±0.12‰ and ±0.20‰, respectively, (2*σ*). *δ*^13^C and *δ*^18^O results are reported, relative to the VPDP and VSMOW standards, as averages of these duplicate analyses, with error bars that are the 2*σ* s.e. of duplicate averages (Supplementary Table 2 and Supplementary Fig. 1).

### Sr-isotopes

^87^Sr/^86^Sr of rock leachates and residues were determined at the University of Cambridge following ref. 47 (Supplementary Table 2 and Supplementary Fig. 1). The internal standard NBS 987 gave 0.710263±0.000009 (1*σ*) on 158 separate measurements made during the course of these analyses. Strontium blanks were always <250 pg and negligible for the Sr concentration of these samples.

### EMPA and scanning electron microscopy analyses

EMPA of mineral compositions were determined using a Cameca SX-100 electron microprobe, using energy-dispersive spectrometry at the University of Cambridge (15 kV, 10 nA; beam diameter 5 μm) with fayalite, rutile, corundum, periclase and pure Co, Ni, Mn, Cr, Zn and Cu standards (Supplementary Table 4). Spectra were collected with a PGT prism 2,000 ED detector and the data reduced with the PGT excalibur software. Back-scatter electron and secondary electron images were obtained using the JEOL 820 scanning electron microscope in the Department of Earth Sciences, University of Cambridge, with an accelerating voltage of 20 kV and 1 nA beam current.

### Porosity measurements by N_2_ physisorption

The surface area and pore-size distribution of sample NPS-069 was investigated using nitrogen gas adsorption techniques at RWTH Aachen University as stated in ref. 35 (Supplementary Table 3 and Supplementary Fig. 6). Specific surface area and total pore volume were determined from nitrogen gas adsorption at 77.3 K, by means of the static-volumetric method, using a Micromeritics Gemini VII 2390t. Samples were prepared by gentle manual crushing of the supplied core sample, with minimal use of energy. The crushed material was manually sieved, the measurements were performed on the 63–400 μm size fraction.

About 1 g of crushed sample material was outgassed in a Micromeritics VacPrep 061 for 12 h at room temperature and then heated to 130 °C, under vacuum, for at least 12 h. Adsorption was measured at 42 relative pressure steps between 0.01 and 0.995, and desorption at 29 relative pressure points between 0.995 and 0.1. The nitrogen saturation pressure (*P*_0_) was determined separately for each relative pressure point. Sorbate–sorbent equilibrium was assumed when the pressure change over a 10-s interval was <0.01% of the average pressure during the latter interval. The multipoint Brunauer–Emmett–Teller theory (BET) method was applied to quantify the surface area of the analysed sample. A cross-sectional area of the nitrogen molecule of 0.162 nm^2^ was used (ISO 9277:2010). Total pore volume was determined by means of the Gurvich rule at an interpolated relative pressure of 0.995, assuming the density of adsorbed N_2_ equals that of liquid N_2_ at the boiling point (28.8 mol l^−1^). Pore volume and area distribution were calculated by means of the Barrett–Joyner–Halenda method in accordance with the DIN 66,134 norm. The Harkins–Jura equations with Faas correction were used for calculation of the statistical thickness curves. The reported Barrett–Joyner–Halenda data are calculated from the adsorption branch of the isotherms, assuming half of the pores are open at both ends. Although desorption isotherms are generally advised for pore size distribution (PSD) calculations, these do not give useful data for shales and other geological materials because of the very strong overprint by the tensile strength effect of N_2_ at ∼40 Å. Repeated measurements on standard materials demonstrated that the accuracy and precision of the methods described above is better than 2% (2*σ*).

### Small angle neutron scattering

Sample porosity and specific surface area was analysed using SANS and VSANS techniques (Fig. 3, Supplementary Figs 4 and 6, and Supplementary Table 3). Experiments were carried out using the instrument KWS-1 (SANS) and KWS-3 (VSANS) operated by the Jülich Center for Neutron Science at Heinz-Meier-Leibnitz Zentrum in Garching, Germany. Carmel caprock samples were cut parallel to bedding, fixed on quartz glass and polished to a thickness of 200 μm for measurements. Samples were dried at room temperature and measurements performed under ambient pressure and temperature conditions. The target area on the sample was defined by a cadmium mask with an 8 mm diameter window.

For (V)SANS measurements, a collimated neutron beam is elastically scattered by the sample^48^,^49^. Position-sensitive detectors measure the scattering intensity *I(Q)* as a function of the scattering angle, which is defined as the angular deviation from the incident beam. The momentum transfer *Q* (nm^−1^) is related to the scattering angle *θ* by *Q*=(4*π*/*λ*)sin(*θ/*2), where *λ* is the wavelength of the neutrons. Thus, the size range of features accessible with neutron scattering depends on the neutron wavelength *λ* and the range in the scattering angle *θ*. SANS data at KWS-1 were collected at wavelengths of *λ*=0.69 nm with a wavelength distribution of the velocity selector Δ*λ*/*λ*=0.10 (full width at half-maximum). Measurements were performed at sample-to-detector distances of 19.7, 7.7 and 1.7 m, covering a wide *Q*-range of 0.02–2.6 nm^−1^. The detector was a ^6^Li glass scintillation detector with an active area of 60 × 60 cm^2^. VSANS data at KWS-3 were collected at *λ*=1.28 nm, Δ*λ*/*λ*=0.2 and a sample-to-detector distance of 9.5 m, covering a *Q*-range from 0.024 to 0.0016, nm^−1^. As for KWS-1, a ^6^Li scintillation detector was used but with a detector diameter of 9 cm. Hence, pore radii for the combined SANS and VSANS measurements range between 1 nm and 1.5 μm (*r*≈2.5/*Q*). Instrument data analysis and background subtraction was carried out using the QtiKWS software provided by the Jülich Center for Neutron Science http://iffwww.iff.kfa-juelich.de/pipich/dokuwiki/doku.php/qtikws. During background subtraction, the lower pore sizes were cut off at 12 Å, to remove artefacts arising from Bragg scattering from ordered stacking of clay minerals. For the combined SANS and VSANS measurements, this results in scattering intensity *I*(*Q*) (nm^−1^) versus the momentum transfer, *Q* (nm^−1^) relationships on nine samples (Supplementary Fig. 4).

The integral of the scattered intensity in reciprocal space in a two-phase system is the Porod invariant *Q*_inv_ from which the porosity *ϕ* is obtained directly^48^,^50^:


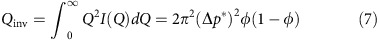


We assumed that the scattering intensity of pore features is directly proportional to the scattering contrast between matrix and pore^48^:


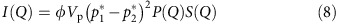


Here, 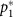 and 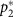 are the coherent scattering length densities (SLDs) for neutrons for the two phases, shale matrix and air (pore), respectively. The terms *P(Q)* and *S(Q)* denote the form and structure factors, for which analytical expressions exist for different geometries of scatterers, including mass and surface fractals^48^. The SLD Δp* of each mineral was calculated as:


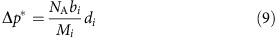


where *b*_*i*_ and *M*_*i*_ are scattering length and atomic mass of the *i*^th^ element in the mineral, *d*_*i*_ is the mass density of the *i*^th^ mineral and *N*_A_ is the Avogadro number. The terms *ϕ* and *V*_p_ are the volume fraction of the dispersed phase and the volume per scatterer, respectively. Scattering length densities were calculated using the U.S. National Institute of Science and technology (NSIT) SLD calculator (http://www.ncnr.nist.gov/resources/activation/). As the scattering contrast between the shale matrix and the pores is large, all scattering is attributed to nano-pore features. SLD values for the shale matrix studied range between 3.2 and 3.4 × 10^−14^ m^−2^; for air, it can be considered to be zero.

The specific surface area of surface fractals scales with the length scale *r* as^48^,^51^:


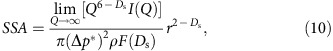


where *ρ* is mass density, *D*_s_ is the surface fractal dimension and


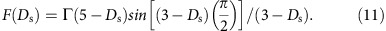


Experimentally determined *I*(*Q*) curves were modelled, after background subtraction, using the polydisperse hard sphere model implemented in the software code PRINSAS^51^. Porosity, pore volume and specific surface area values obtained from this analysis are summarized in Supplementary Table 2; intensity, *I*(*Q*) and pore frequency distribution *f(r)* plots for all nine samples analysed are shown in Supplementary Fig. 4. Effective diffusion values *D*_e_ (m^2^ s^−1^) were calculated from the fractal dimensions derived from the (V)SANS data (Fig. 3c). Typically, *D*_e_ is related to the diffusion coefficient in water, *D*_w_ and the diffusion accessible porosity, *ϕ*, by


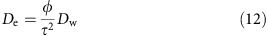


where *τ* is the tortuosity. Using fractal dimensions derived from (V)SANS measurements; however, the effective diffusion coefficient is related to the diffusion accessible porosity by an analogue empirical power law formulation of the form:


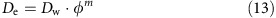


where *m* is an empirical exponent. In natural porous media the power law form (equation 13) is indicative of the fractal geometry of the pore–solid interface. These fractal dimensions of the pore surface and pore volume can be quantified from (V)SANS analysis. In such a sinuous capillary bundle model, the sinuous nature of the bundle reflects the roughness of the pore–solid interface, with fractal properties within some scale limits. The power law form (equation (13)) can thus be related to the porosity of a volume element of size *l*_2_, between the lower and upper limits of the fractal region, *l*_1_ and *l*_2_, and to the pore volume fractal *D*_v_, as


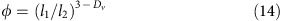


where, for embedded dimensions of three^52^, 2≤*D*_v_≤3. The tortuosity(Fig. 3c) of the fractal bundle in a volume element is related to the fractal dimensions of the pore–solid surface, *D*_s_, and pore volume, *D*_v_, as


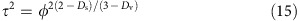


Substituting equation (15) into equation (12) gives


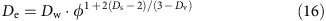


where the fractal dimensions of the pore surface and pore volume can be quantified from (V)SANS analysis as the slope of *Q* versus *I(Q)* and *r* versus *f(r)*, respectively, in the *Q*-range 10^−4^ Å^−1^≤Q≤10^−2^ Å^−1^.

### Hydraulic conductivity measurements

A section of core from a red siltstone unit of the lower Carmel Formation (depth: 180.4–180.7 m) was selected during drilling and preserved under mechanical confinement on-site for later analysis in the laboratory. Intrinsic permeability of subsections of the core interval were measured in a bespoke permeameter designed and built by the British Geological Survey. Each core plug sample was carefully manufactured on a machine lathe to minimize damage and desaturation, and to produce samples of known dimension. Samples were then placed inside a single closure pressure vessel and subject to *in situ* stress and porewater pressure conditions, to hydrate the sample before testing. Permeability was measured by the application of a fixed pressure gradient across each core, while simultaneously measuring flux in and out of the sample. Volumetric flow rates into and out of the core were controlled or monitored using a pair of ISCO-260, Series D, syringe pumps operated from a single digital control unit. The position of each pump piston is determined by an optically encoded disc graduated in segments equivalent to a change in volume of 16.6 nl. Movement of the pump piston is controlled by a micro-processor that continuously monitors and adjusts the rate of rotation of the encoded disc using a DC motor connected to the piston assembly via a geared worm drive. This allows each pump to operate in either constant pressure or constant flow modes. A programme written in LabVIEW elicits data from the pump at pre-set time intervals. Testing was performed in an air-conditioned laboratory at a nominal temperature of 20 °C.

Darcy's law gives the following relationship for the conductivity, *K*,


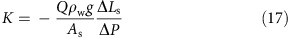


where *Q* is the steady-state flow (m^3^ s^−1^), *p*_w_ is the density of water (kg m^−3^), *g* is the acceleration due to gravity (m s^−2^), *A*_s_ is the cross-sectional area of the test sample normal to flow (m^2^), Δ*L*_s_ is the sample length (m) and Δ*P* is the pressure drop along the sample (Pa). The equivalent permeability term (*k*) is given by


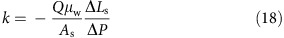


where *μ*_w_ is the viscosity of water (1.002 × 10^−3^ Pa s). Measurements on the four selected core intervals gave permeabilities between 5 × 10^−22^ and 1.2 × 10^−21^ m^2^ with an average value of 9.1 × 10^−22^ m^2^ (1σ=2.3 × 10^−22^ m^2^).

### Reaction rates for dolomite and haematite

Dolomite dissolution rates are given by Pokrovsky *et al*.^53^ who modelled experimental determinations of dolomite dissolution rates by various pH-dependent surface processes. At the intermediate pH conditions and CO_2_ partial pressures in the cap rock, the predicted reaction rates are between 5 × 10^−7^ and 10^−5^ mol m^−2^ s^−1^. dos Santos Afonso and Stumm^41^ measured haematite dissolution rates as a function of pH and H_2_S concentrations. For the pH and estimated partial pressures of H_2_S in the caprock, the predicted dissolution rates lie between 10^−10^ and 10^−8^ mol m^−2^ s^−1^.

### Reactive transport modelling with analytical solution

Advective flow rates, (*ω*_0_*ϕ*) where *ω*_0_ is fluid velocity and *ϕ* porosity, through the caprock are estimated to be <6 × 10^−12^ m s^−1^ given permeabilities of ∼10^−21^ m^2^ and a piezometric pressure gradient of ∼10^4^ to 4 × 10^6^ Pa m^−1^ taking the hydraulic head in the Navajo^54^ either across the whole Carmel Formation or the 16 cm basal seal. With effective diffusivities (*D*_e_) of components in the fluid phase of ∼10^−11^ to 10^−12^ m^2^, the Péclet number (*ω*_0_*ϕ*h/*D*_e_) for advective–diffusive transport of a compatible component would be in the range 10^−7^ to 10^−2^, values that imply advective transport is negligible consistent with the analysis of the caprock transport. We therefore model reactive transport in the caprock driven only by diffusion.

The equation describing one-dimensional diffusive transport with mineral reaction of solute concentration *C* (mol m^−3^) with respect to distance *x* (m), time *t* (s), effective diffusivity *D* (m^2^ s^−1^), porosity *ϕ*, tortuosity, *τ*^2^, mineral reaction rate *k*_*f*_ (m s^−1^) and surface area *α* (m^2^ m^−3^) can be written as^39^


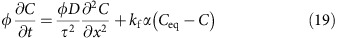


where (*C*_eq_*−C*) describes a linear dependence of the reaction rate to the difference between the solute component concentration, *C* (mol m^−3^) and its concentration at equilibrium, *C*_eq_, with the reacting mineral. The mineral molar concentration in the rock, *ϕ*_s_ (mol m^−3^), then varies according to


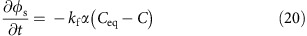


Making the transformation to dimensionless variable by


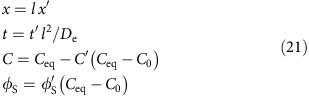


where *l* (m) is a transport distance taken as the displacement of the reaction front, *D*_e_ is the effective diffusion coefficient *ϕD*/*τ*^2^ and *C*_0_ is solute concentration in the input fluid. Substituting equation (21) into equations (19) and (20) gives


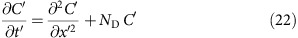



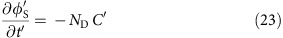


dependent on the dimensionless constant, the Damköhler number *N*_D_, defined by


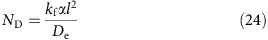


For reaction rates of dolomite (*k*_R_) between 10^−6^ and 10^−8^ mol m^−2^ s^−1^ (note *k*_f_=*k*_R_*V*_S_ where *V*_S_ is the molar volume of dolomite) and diffusion coefficients in the range 1 × 10^−11^ to 5 × 10^−12^ m^2^ s^−1^, Damköhler numbers are in the range 5 × 10^7^ to 9 × 10^9^ and fluids will be effectively in local equilibrium with minerals. For haematite with reaction rates in the range 10^−8^ to 10^−12^ mol m^−2^ s^−1^, Damköhler numbers are in the range 2 × 10^3^ to 3 × 10^7^ and haematite will be close to equilibrium wherever it is in contact with CO_2_ transported by diffusion.

On initiation of diffusion, dissolution of the reactive mineral takes place at a rate, which decreases away from the inlet such that the mineral is exhausted after time *τ*_0_ (s) given by


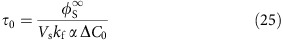


or in dimensionless time,


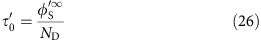


At times greater than *τ*_0_, the reactive mineral is exhausted over distances *x*≤*l*. Where concentrations in the fluid are small compared with those in the solid and porosities are small, the approximate quasi-stationary state solutions to equations (19) and (20) are valid^39^. Solutions for fluid compositions and solid mineral modes, assuming mineral surface area remains constant, following ref. 39, in dimensionless coordinates for *τ*_0_>0, are (note *l*′=1)


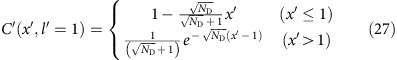



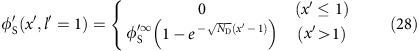


where Δ*C*_0_=*C*_eq_−*C*_0_. Δ*C*_0_ for Fe_2_O_3_ in reaction (1), between the inlet solution and a solution at equilibrium with haematite, has been calculated using PHREEQC^42^ as 2.95 mol m^−3^ for an inlet solution with the average composition of the fluids sampled downhole in the Navajo Sandstone, with a range from 1.9 to 3.95 mol m^−3^ reflecting the range in pH of 5.3–5.1 of the downhole fluids^23^. It is noteworthy that the Damköhler number, *N*_D_=(*ql*)^2^, with *q* (m^−1^) defined by Lichtner^39^ as the exponential constant giving the length scale over which fluid returns to equilibrium with the initial mineralogy. The time for the reaction front to migrate to distance, *l*, (*l*′=1) is given by solution of


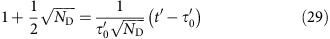


or in terms of dimensional constants


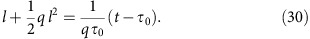


### Multicomponent reactive transport modelling

PHREEQC modelling was conducted using the llnl.dat thermodynamic data base thermo.com.V8.R6.230 (ref. 42). A 15-cm-long one-dimensional reactive–diffusive model comprising 30 cells of 5 mm length was constructed. The initial mineralogy (mol l^−1^) was calculated from quantitative mineralogy determined by XRD, using pore volumes measured from SANS of the unaltered portion of the caprock. The invading pore fluid chemistry was based on analyses of the reservoir fluids (Supplementary Table 4) and the initial caprock pore fluid chemistry was taken to be a fluid in equilibrium with the caprock mineralogy, with pCO_2_, pO_2_ and salinity estimates for typical Jurassic marine shales. The initial redox state of the invading fluid was defined using the SO_4_^2−^/H_2_S redox couple. Models were run assuming local fluid–mineral equilibrium. A constant *D*_e_ value of 5 × 10^−12^ m^2^ s^−1^ was used for all aqueous species. The model timescale was 125,000 years with a time step of 7 days. The thermodynamic database was supplemented with data for Fe-dolomite (CaMg_0.9_Fe_0.1_(CO_3_)_2_) calculated using ideal mixing laws and the thermodynamic data for ankerite taken from ToughReact TherAkin8.dat.

A second model was constructed comprising two 25,000 year pulses of CO_2_-charged brine injection separated by a 75,000 year period where a hypothetical CO_2_-poor brine was diffused into the model cells (Fig. 4b). The durations of each pulse were chosen to mimic the timescale of periodic degassing of CO_2_ previously document for the local fault systems^26^.

### Data availability

The authors declare that the data supporting the findings of this study are available within the article and its Supplementary Information files.

## Additional information

**How to cite this article:** Kampman, N. *et al*. Observational evidence confirms modelling of the long-term integrity of CO_2_-reservoir caprocks. *Nat. Commun.* 7:12268 doi: 10.1038/ncomms12268 (2016).

## Supplementary Material

Supplementary Information Supplementary Figures 1-6, Supplementary Tables 1-4 and Supplementary References
