# Supplementary material for: Observational evidence confirms modelling of the long-term integrity of CO2-reservoir caprocks
Source: Nat Commun. 2016 Jul 28;7:12268. doi: 10.1038/ncomms12268 (PMC4974477; doi:10.1038/ncomms12268)
Supplement: Supplementary Information — Supplementary Figures 1-6, Supplementary Tables 1-4 and Supplementary References [file ncomms12268-s1.pdf]

Supplementary Information: Figures

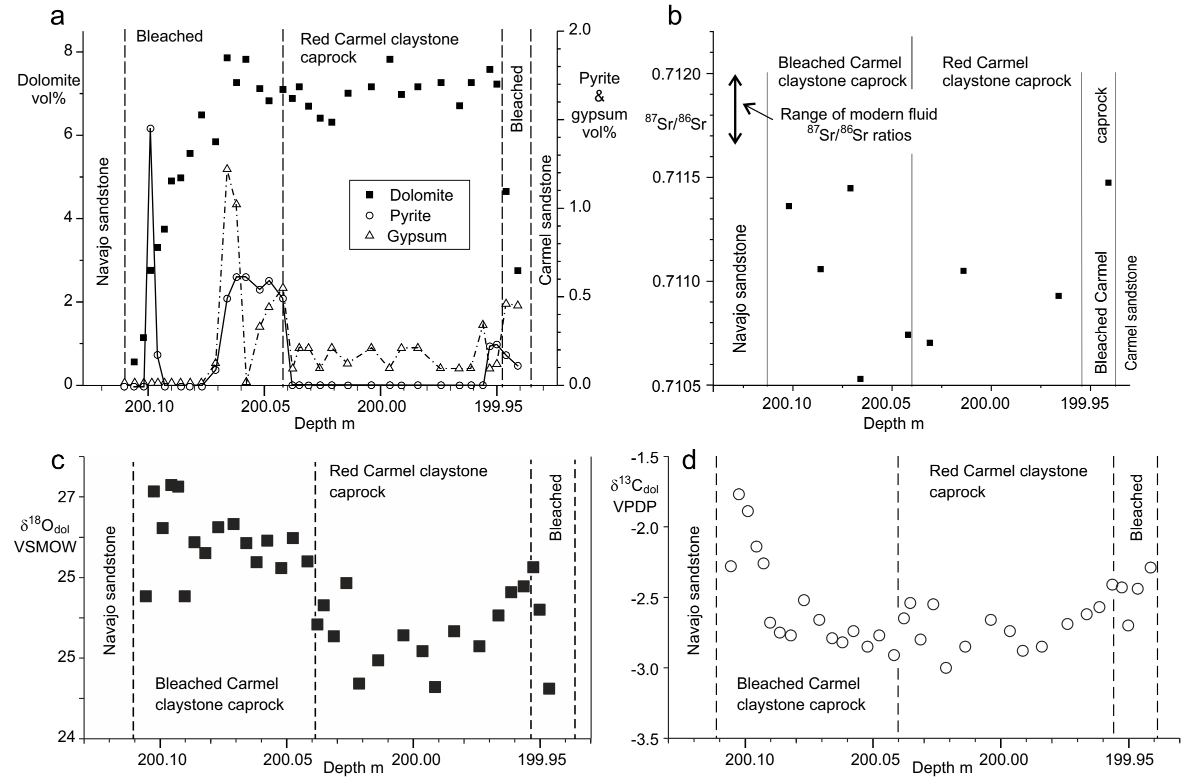

Supplementary Fig. 1. **Mineral mode and isotopic profiles across the 16 cm caprock.** (**a**) Profiles for dolomite (both Mg and Fe-bearing), pyrite and gypsum volume percentages. (**b**) $^{87}Sr/^{86}Sr$ ratio of the carbonate fraction in the caprock (Supplementary Table 2) compared to range of $^{87}Sr/^{86}Sr$ ratios of present day fluids in the underlying Navajo sandstone[1]. Carbonate leaches analysed from the Navajo sandstone drill core have $^{87}Sr/^{86}Sr$ ratios of 0.7129 and 0.7150. A longer time-record of fluid $^{87}Sr/^{86}Sr$ ratios in travertines at Green River[2] ranges from 0.7116 to 0.7134. (**c**) $\delta^{18}O$ dolomite and (**d**) $\delta^{13}C$ of dolomite profiles. The Sr-, oxygen- and carbon-isotopic profiles all indicate that re-precipitation of dolomite accompanies dissolution of dolomite consistent with petrographic evidence for replacement of Mg-rich dolomites with more Fe-rich dolomite.

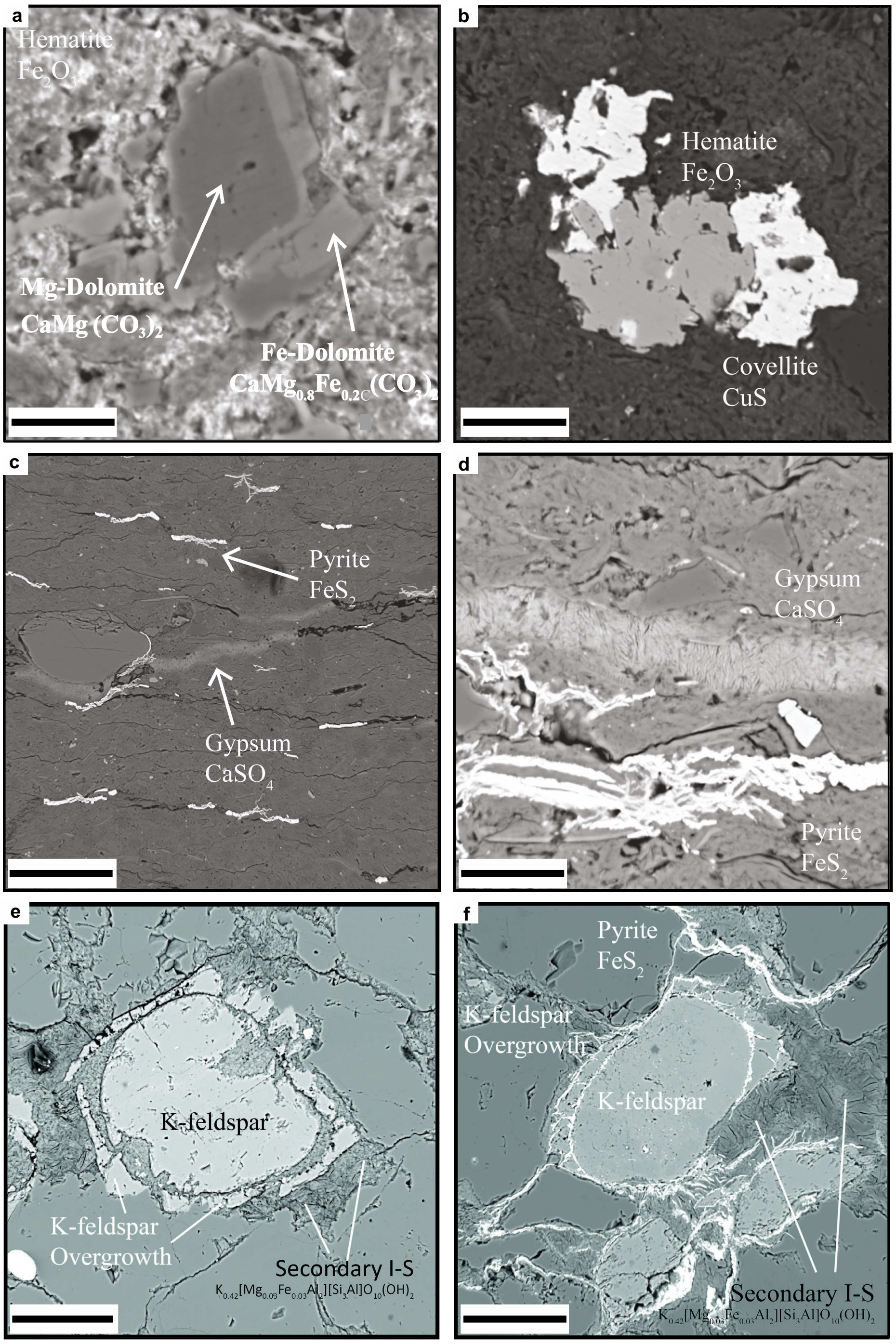

Supplementary Fig. 2. **Images of Carmel caprock.** Secondary electron (SE) and back-scatter electron (BSE) SEM images from the Carmel Formation caprock profile. (**a** & **b**). Images taken downstream of the reaction front, adjacent to the haematite dissolution front boundary. (**a**) Intergrowths of haematite and covellite. Scale bar 10 μm. (**b**) Fe-rich dolomite (light) rimming earlier Mg-rich dolomite. Scale bar 20 μm. (**c** & **d**) BSE and SE SEM images of pyrite and gypsum precipitation upstream of the haematite dissolution front. (**c**) Pyrite (bright) and gypsum (pale vein) distributed throughout the clay matrix of the altered caprock. Scale bar 100 μm. (**d**) Close-up of pyrite and gypsum veins. Scale bar 10 μm. (**e** & **f**) BSE SEM images of clay and pyrite precipitation close to the caprock-reservoir interface. (**e**) Corroded K-feldspar grains, overgrown with secondary K-feldspar, replaced by illite. Secondary Illite also fills primary pore space. Scale bar 100 μm. (**f**) K-feldspar overgrown by new K-feldspar rim and illite which also fills pore space. Pyrite (bright) also mantles grain boundaries. Scale bar 40 μm.

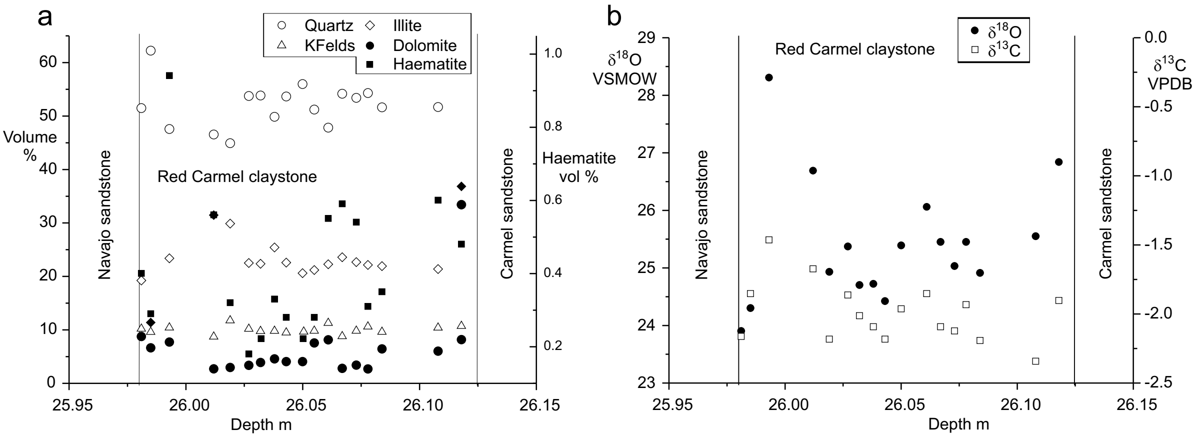

Supplementary Fig. 3. **Mineral modes in CO_2-free Carmel Formation.** (**a**) Variation in mineral modes (volume %) and (**b**) oxygen- and carbon-isotopic ratios of dolomite across the basal claystone in the Carmel Formation in the BH1 drill core which penetrated the Carmel and Navajo Formations in the hanging wall of the Bighole fault 33 km northwest of Green River[3]. No systematic changes in mineral modes or isotopic compositions are seen at this locality where there is no evidence of CO_2 injection.

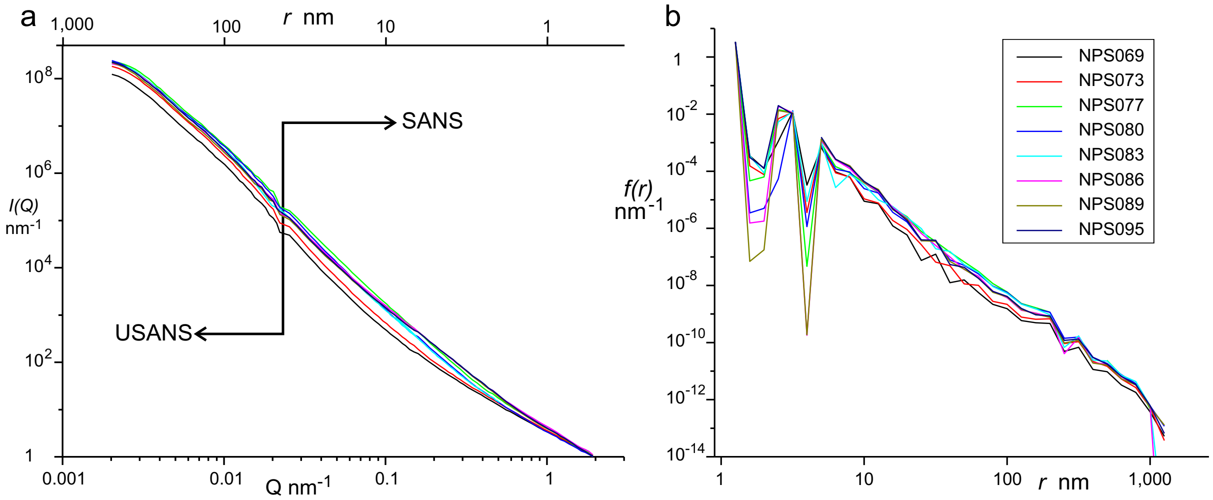

Supplementary Fig. 4. **Small angle neutron scattering data.** (**a**) Scattering intensity *I(Q)* versus the scattering vector, *Q*, for the SANS and (V)SANS data on nine samples (depths in Supplementary Table 1). (**b**) Pore volume distribution, *f(r)*, as a function pore radius, *r*, from the SANS and (V)SANS data.

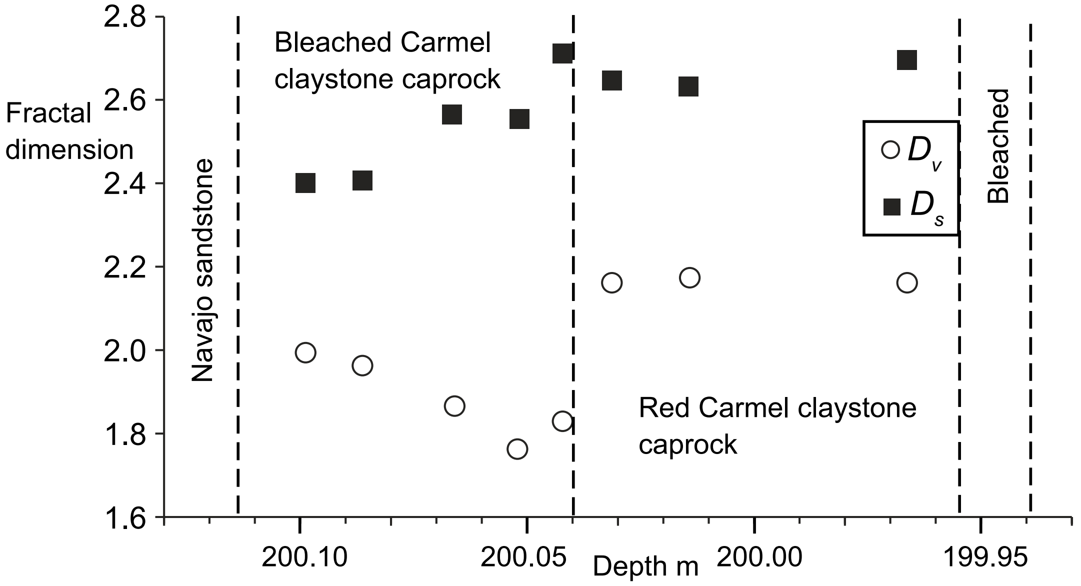

Supplementary Fig. 5. Variation in the dimensionless surface fractal dimension (D_s) and pore volume fractal (D_v) from the SANS/(V)SANS data across the caprock.

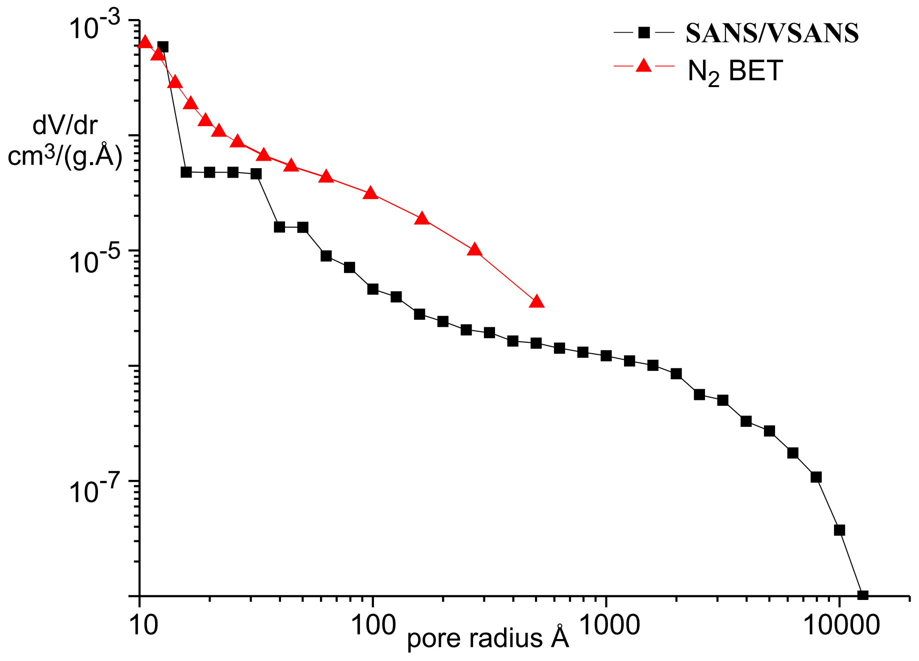

Supplementary Fig. 6. Comparison of porosity distribution as a function of pore size from N_2-BET data compared with that calculated from SANS/(V)SANS data for sample NPS-069.

Supplementary Tables

Supplementary Table 1. Caprock mineral modes (wt%) determined by XRD

Sample	Depth metres	Quartz	K-feldspar	Illite	Dolomite	Siderite	Haematite	Anatase	Pyrite	Gypsum	Total
NPS101	199.94	13.3	6.5	74.2	2.9	0.1	1.8	0.7	0.2	0.4	100.1
NPS100	199.95	10.2	6.1	74.6	4.9	0.1	2.7	0.8	0.3	0.4	100.1
NPS099	199.95	3.9	3.1	80.0	7.5	0.1	4.0	0.8	0.4	0.1	99.9
NPS098	199.95	4.6	2.5	80.0	7.9	0.1	3.8	0.8	0.4	0.0	100.1
NPS097	199.96	4.7	2.6	78.1	8.9	0.2	4.1	0.7	0.3	0.3	99.9
NPS096	199.96	4.9	3.2	78.8	7.7	0.1	4.2	0.7	0.3	0.1	100.0
NPS095	199.97	4.8	3.3	79.9	7.0	0.1	3.8	0.7	0.3	0.1	100.0
NPS094	199.97	5.9	2.8	78.6	7.7	0.1	3.7	0.7	0.3	0.1	99.9
NPS093	199.98	5.4	2.7	79.6	7.6	0.1	3.5	0.7	0.3	0.2	100.1
NPS092	199.99	5.5	2.7	79.6	7.3	0.1	3.8	0.7	0.3	0.2	100.2
NPS091	200.00	5.9	3	77.9	8.3	0.1	3.6	0.7	0.3	0.1	99.9
NPS090	200.00	6	2.8	78.2	7.6	0.1	4.0	0.8	0.3	0.2	100.0
NPS089	200.01	5.5	2.7	79.2	7.3	0.1	4.1	0.7	0.3	0.1	100.0
NPS088	200.02	6.5	3	78.5	6.7	0.1	4.0	0.6	0.3	0.2	99.9
NPS087	200.03	5.8	3	79.2	6.8	0.1	3.9	0.7	0.3	0.1	99.9
NPS086	200.03	6.3	2.3	79.6	7.0	0.1	3.5	0.7	0.3	0.2	100.0
NPS085	200.04	6.1	2.6	79.2	7.6	0.1	3.1	0.8	0.3	0.2	100.0
NPS084	200.04	6.2	2.8	79.6	7.3	0.1	2.9	0.8	0.3	0.0	100.0
NPS083	200.04	5.8	3.2	81.1	7.7	0.0	0.0	0.8	0.9	0.5	100.0
NPS082	200.05	5.8	2.6	81.7	7.4	0.1	0.2	0.7	1.1	0.4	100.0
NPS080	200.05	6.1	3.1	80.7	7.7	0.1	0.3	0.8	1.0	0.3	100.1
NPS079	200.06	7	2.8	79.7	8.3	0.1	0.2	0.7	1.1	0.0	99.9
NPS078	200.06	5.9	3.2	80.3	7.7	0.1	0.2	0.7	1.1	0.9	100.1
NPS077	200.07	6	3.2	79.4	8.5	0.1	0.0	0.8	0.9	1.1	100.0
NPS076	200.07	5.1	2.6	84.8	6.2	0.1	0.0	0.9	0.2	0.1	100.0
NPS075	200.08	6	2.7	83.2	7.0	0.1	0.1	0.9	0.0	0.0	100.0
NPS074	200.08	5	2.8	85.2	5.9	0.1	0.1	0.8	0.0	0.0	99.9
NPS073	200.09	5.2	2.8	85.7	5.3	0.0	0.0	0.9	0.0	0.0	99.9
NPS072	200.09	5.2	2.6	86.0	5.2	0.1	0.1	0.9	0.0	0.0	100.1
NPS071	200.09	5.3	2.8	87.0	4.0	0.1	0.1	0.9	0.0	0.0	100.2
NPS070*	200.10	5.1	3.3	86.1	3.5	0.0	0.0	0.9	0.3	0.0	100.1
NPS069	200.10	8.1	3.5	82.0	2.9	0.0	0.0	0.9	2.6	0.0	100.0
NPS068*	200.10	49.2	10.6	37.0	1.2	0.0	0.0	0.1	0.0	0.0	100.0
NPS067	200.11	26	8.9	63.9	0.6	0.0	0.0	0.6	0.0	0.0	100.0

*Sample NPS070 also contained 0.8 wt% albite and 0.1 % apatite and sample NPS068 also contained 1.9% calcite

Supplementary Table 2. | Isotopic compositions of caprock samples

Sample ID	Depth	$\delta^{18}O_{carb}$	$\delta^{13}C_{carb}$	$^{87}Sr/^{86}Sr$	$^{87}Sr/^{86}Sr$
	meters	‰ VSMOW	‰ VPDB	carbonate	silicate
NPS107				0.713171	
NPS101	199.94	22.75	-2.29	0.711477	
NPS100	199.95	24.62	-2.44		
NPS099	199.95	25.60	-2.7		
NPS098	199.95	26.13	-2.43		
NPS097	199.96	25.89	-2.41		
NPS096	199.96	25.82	-2.57		
NPS095	199.97	25.53	-2.62	0.710931	
NPS094	199.97	25.15	-2.69		
NPS093	199.98	25.33	-2.85		
NPS092	199.99	24.64	-2.88		
NPS091	200.00	25.09	-2.74		
NPS090	200.00	25.28	-2.66		
NPS089	200.01	24.97	-2.85	0.711052	
NPS088	200.02	24.68	-3.00		
NPS087	200.03	25.93	-2.55		
NPS086	200.03	25.27	-2.8	0.710705	0.715679
NPS085	200.04	25.65	-2.54		
NPS084	200.04	25.42	-2.65		
NPS083	200.04	26.20	-2.91	0.710744	
NPS082	200.05	26.49	-2.77		
NPS080	200.05	26.12	-2.85		
NPS079	200.06	26.46	-2.74		
NPS078	200.06	26.19	-2.82		
NPS077	200.07	26.43	-2.79	0.710531	
NPS076	200.07	26.66	-2.66	0.711449	
NPS075	200.08	26.62	-2.52		
NPS074	200.08	26.30	-2.77		
NPS073	200.09	26.44	-2.75	0.711059	0.715136
NPS072	200.09	25.77	-2.68		
NPS071	200.09	27.13	-2.26		
NPS070	200.10	27.15	-2.14	0.719664	
NPS069	200.10	26.61	-1.89		
NPS068	200.10	27.06	-1.77	0.711363	0.714860
NPS067	200.11	25.77	-2.28		

Supplementary Table 3. Petrophysical data for caprock samples and parameters describing pore structure

	Depth	SANS surface area	SANS cumulative pore volume x10^3	SANS porosity	D_v	D_s	m	τ^2	D_e
	m	m^2/g	cm^3/g	%					$m^2.s^{-1}$
NPS095	199.966	34.6	38.6	9.99	2.16	2.69	2.64	44	4.5E-12
NPS089	200.014	30.4	35.9	9.38	2.17	2.63	2.52	36	5.2E-12
NPS086	200.031	37.8	40.2	10.33	2.16	2.64	2.53	32	6.4E-12
NPS083	200.042	30.4	39.2	9.94	1.83	2.71	2.21	16	1.2E-11
NPS080	200.052	35.0	40.9	10.4	1.76	2.55	1.88	7	2.8E-11
NPS077	200.066	34.7	47.4	11.78	1.86	2.56	1.99	8	2.9E-11
NPS073	200.086	38.8	35.8	8.96	1.96	2.40	1.77	6	2.8E-11
NPS069	200.099	33.1	27.8	7.38	1.99	2.40	1.78	8	1.9E-11

Supplementary Table 4: Electron-microprobe mineral compositions and fluid compositions

Sample	*n*		$CaCO_3$	$MgCO_3$	$FeCO_3$	Mn	Sr
			------------mol%		----------	----- *ppm*	----
NPS088 red claystone	27	Average	51.0	44.9	3.7	2067	149
		1σ	1.3	5.1	4.1	1920	245
		Ankerite	50.8	34.8	13.5	4744	0
		Dolomite	51.5	48.1	0.3	477	325
NPS084 red claystone	32	Average	51.0	44.5	4.1	2337	135
		1σ	1.1	5.7	4.9	2510	169
		Ankerite	49.0	33.4	13.9	8418	729
		Dolomite	52.9	47.0	0.1	40.7	237
NPS082 bleached claystone	14	Average	50.8	47.0	1.8	2126	85
		1σ	1.8	5.6	4.8	2626	154
		Ankerite	52.5	28.2	18.3	4987	243
		Dolomite	50.4	49.3	0.1	1054	0
NPS075 bleached claystone	20	Average	50.7	48.5	0.6	967	235
		1σ	1.2	1.3	0.3	792	240
		Ankerite	51.6	46.6	1.4	1842	186
		Dolomite	49.9	49.9	0.2	173	79

Mineral Phase	*n*		Fe	Cu	S	Mn	As	Co	Ni
			----------- *atomic %*		----------	-------------- *ppm*			-----------
Chalcopyrite	9	Average	24.4	24.6	50.3	174	892	103	162
		1σ	0.6	0.6	0.3	179	430	80	152
Pyrite	19	Average	33.2	0.0	66.3	102	626	77	108
		1σ	0.3	0.0	0.5	90	190	75	56
Covellite	2	Average	1.0	47.2	50.8	112	92	B.D.	57
		1σ	0.2	0.5	0.4	90	13	0	17

Mineral Phase	*n*		Si	Al	Ti	Al	Fe	Mg	Mn	Ca	Na	K
			----------------	*IV*	------------	------------	*VI*	----------	----------	----	*interlayer*	----
Matrix Illite	24	Average	3.45	0.53	0.026	1.47	0.26	0.33	0.002	0.06	0.03	0.74
		1σ	0.20	0.20	0.012	0.12	0.06	0.09	0.002	0.10	0.01	0.06
Pore Filling Illite	9	Average	3.02	0.97	0.001	2.12	0.03	0.03	0.000	0.01	0.04	0.42
		1σ	0.10	0.10	0.001	0.08	0.01	0.01	0.001	0.01	0.01	0.12

Fluid compositions	pH	Al^{3+}	Ca^{2+}	Mg^{2+}	K^+	Na^+	SiO_2	Cl^-	SO_4^{2-}	H_2S	Alk	DIC
						mmol/L						
Caprock Pore Water	7.52	0.00	13.6	1.63	1.43	23.9	0.06	30.4	23.9	0.00	0.76	0.8
CO_2-Reservoir Brine	5.1	0.01	24.1	10.1	7.2	112.5	0.1	84.9	20.7	0.5	64.0	884
CO_2-poor Reservoir Brine	7.09	0.01	24.1	10.1	7.2	112.5	0.1	145.6	20.7	0.5	2.5	2.5

Supplementary references

1 Kampman, N. *et al.* Drilling and sampling a natural CO_2 reservoir: Implications for fluid flow and CO_2-fluid–rock reactions during CO_2 migration through the overburden. *Chemical Geology* **369**, 51-82 (2014).

2 Kampman, N. *et al.* Pulses of carbon dioxide emissions from intracrustal faults following climatic warming. *Nature Geoscience* **5**, 352-358, (2012).

3 Shipton, Z. K., Evans, J. P., Robeson, K. R., Forster, C. B. & Snelgrove, S. Structural heterogeneity and permeability in faulted eolian sandstone: Implications for subsurface modeling of faults *AAPG Bulletin* **86**, 863-883 (2002).
